# Extended Detection Window for Gamma-Hydroxybutyrate in the Urine of an Elderly Woman

**DOI:** 10.1093/jat/bkad014

**Published:** 2023-02-27

**Authors:** J A Berg, R Rajendiran, T T Serkland, C B Methlie, E I Hallin, C L Stokes

**Affiliations:** Department of Medical Biochemistry and Pharmacology, Haukeland University Hospital, Jonas Lies v 65, Bergen 5021, Norway; Family Physician, Arkaden Legekontor, Arkaden Legekontor, Sartorvegen 12, Straume 5353, Norway; Department of Medical Biochemistry and Pharmacology, Haukeland University Hospital, Jonas Lies v 65, Bergen 5021, Norway; Department of Medical Biochemistry and Pharmacology, Haukeland University Hospital, Jonas Lies v 65, Bergen 5021, Norway; Department of Medical Biochemistry and Pharmacology, Haukeland University Hospital, Jonas Lies v 65, Bergen 5021, Norway; Department of Medical Biochemistry and Pharmacology, Haukeland University Hospital, Jonas Lies v 65, Bergen 5021, Norway

## Abstract

Gamma-hydroxybutyrate (GHB) is a central nervous system depressant that has gained popularity as an illicit recreational drug. We describe a case of an elderly woman who was found unconscious in her home. The paramedics initially suspected an intracranial incident. A head computed tomography was negative, as was the initial urinary drug screening. The diagnosis of GHB intoxication was made with the detection of GHB in a urine sample obtained 28–29 hours after the assumed time of intake. Our case underscores the importance of considering drug testing in a broad range of patients and shows that elderly patients may have an extended detection window of GHB.

## Introduction

Gamma-hydroxybutyrate (GHB) is an endogenous substance closely related to the inhibitory neurotransmitter gamma-amino butyric acid. Synthetic GHB was first produced in an attempt to create a drug with sedative properties to be used as an anesthetic and has later been introduced as a therapeutic drug for narcolepsy treatment, more specifically for cataplexia (Xyrem^®^). However, it is now mostly recognized as a recreational drug (1). Because of a short elimination half-life, the detection window of GHB is narrow in both blood and urine, and GHB intoxications can therefore be difficult to diagnose. The literature mostly describes the pharmacokinetics (PK) of GHB in young adults after the intake of therapeutic doses (2). The PK in high doses and in the elderly is inadequately studied. Some have indicated that one can reach enzymatic saturation after the intake of supratherapeutic doses of GHB, giving rise to an increase in detection time (3). According to the summary of product characteristics (SPC) of Xyrem^®^, there is very limited experience with sodium oxybate in the elderly. Furthermore, the SPC states that “in a limited number of patients greater than the age of 65 years, the PK of sodium oxybate was not different compared to patients younger than 65 years of age” (4). It is reasonable to assume that these patients were administered the drug in therapeutic doses. The PK after the high-dose intake in elderly patients is, to our knowledge, not described in the literature.

## Method

### Sample preparation

The patient’s urine sample, serum sample, and calibration standards and quality controls (20 µL) were mixed with a water solution (180 µL) containing internal standard, 3.33 mg/L γ-hydroxy butyric acid-d_6_ (GHB-d_6_, 9859.4 K-ME, Chiron, Trondheim, Norway), 0.40 mg/L ethyl-β-D-glucuronide-d_5_ (EtG-d_5_, E-063, Cerilliant, TX, USA) and 0.34 mg/L ethylsulfate-d_5_ dissolved (EtS-d_5_, ETS-979-NA-1 LM, Lipomed, Arlesheim, Switzerland) in 0.1% formic acid (1.00264, Merck, Germany). The serum sample was treated the same way as the urine sample but with an additional ultracentrifugation step through a 30-kDa MWCO Amicon Ultra spin filter (Merck, Germany) before the mass spectrometry (MS) analysis. The calibration standards and quality controls were made by spiking “blank” urine with γ-hydroxy butiric acid (GHB, 9858.4-10 K-ME, Chiron, Trondheim, Norway), ethyl-β-D-glucuronide (EtG, C1626.8 K-ME, Chiron, Trondheim, Norway) and ethylsulfate (EtS, ETS-972-NA-1 LM, Lipomed, Arlesheim, Switzerland). The “blank” urine could contain endogenous GHB but with a concentration lower than half of the lowest calibrator standard. Standard concentrations of GHB (5.16, 12.0, 24.1, 286 and 2,870 mg/L), EtG (0.105, 0.246, 0.492, 5.86 and 58.6 mg/L) and EtS (0.053, 0.123, 0.246, 2.93 and 29.3 mg/L) and quality control concentrations of GHB (7.53, 12.6 and 100 mg/L), EtG (0.150, 0.251 and 2.00 mg/L) and EtS (0.075, 0.126 and 1.00 mg/L) were used. Note that the calibrator, quality control samples and patient samples will be diluted when mixed with the internal standard, resulting in a final concentration of the GHB internal standard of 3.0 mg/L, corresponding to an analyte concentration between the third and fourth calibrator levels. The final concentrations of the five GHB calibrators were 0.52, 1.2, 2.4, 29 and 287 mg/L.

### Liquid chromatography coupled to tandem mass spectrometry method

The samples were loaded (1 µL) onto an ExionLC AD UHPLC system (SCIEX Concord, ON, Canada), with a Kinetex 2.6 µm XB-C18, 100 × 2.1 mm column (Phenomenex, CA, USA), connected to a QTRAP 6500+ mass analyzer (SCIEX, Concord, ON, Canada). Mobile phases used were A, 0.1% formic acid (Merck, Germany), and B, acetonitrile with 0.1% formic acid (Merck, Germany). The flow rate was 0.6 mL/min, and the elution gradient was starting at 100% A to 85% after 1 min, 10% at 1.1 min and back to 100% at 1.7 min with a total run time of 2.5 min. The mass analyzer was in a negative mode with a declustering potential of −40 V, entrance potential of −10 V, collision cell exit potential of −8 V and mass transitions of 20 ms dwell time at 103.04/85.03 Da, CE −13 V for GHB quantifier and 103.04/101.02, CE −15 V for GHB qualifier and 109.08/61.06 and CE −20 V for GHB-d_6_. The peak area ratio between the analyte and internal standard was used to determine the concentration of GHB in the unknown samples.

### Method validation

**Figure 1.**
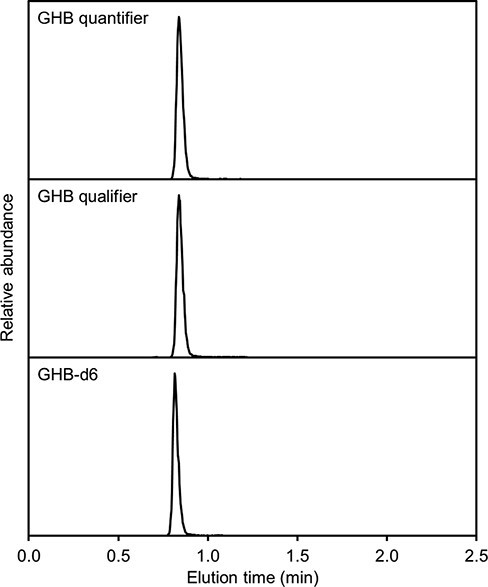
Extracted-ion chromatograms of GHB quantifier, qualifier and the internal standard GHB-d_6_ from LC--MS-MS analysis of the patient’s urine sample.

The method has been validated according to the International Organization for Standardization standard 15189. The total variation and accuracy were tested >10 days, in urine samples spiked at three concentrations 0.75, 1.3 and 10 mg/L (1.5, 2.5 and 20 times the concentrations of the lowest calibrator at 0.52 mg/L), with 100 analytical measurements in each of the three controls. The three controls had a coefficient of variation (CV%) of 1.7%, 1.6% and 1.9%, respectively. No carryover was seen in a blank sample analyzed after a sample with the highest calibrator (287 mg/L). The limit of detection for GHB was 0.17 mg/L, and the limit of quantification (LOQ) was 2.0 mg/L. The CV for LOQ was 6.3%. Linearity was tested with square, power and linear curve fitting with and without weighting on data from liquid chromatography--tandem mass spectrometry (LC--MS-MS) analysis of the calibration standards (*n* = 8). Using a square fit with weighting of 1/x^2^ resulted in the highest accuracy for quality control samples. Ten patient urine samples were tested for matrix effects, giving a factor of 1.09 ± 0.045 after correction with internal standard. The stable retention time for GHB was confirmed, demanding a variation within 1%. The selectivity of the method was assessed by examining a wide selection of pharmaceuticals and drugs of abuse and the endogenous compounds alpha- and beta-hydroxybutyrate, altogether 114 different compounds. None of the compounds showed interference. Peak area ratios between quantifier and qualifier ions for GHB samples were required to be within 30% of the average quantifier/qualifier ion ratio from the five spiked samples used as a standard. The urine sample in the present case (Figure 1) had a quantifier/qualifier ion ratio of −4.9% compared to the calibrator samples. The relative retention time for the GHB peak was +0.17%.

To evaluate the method performance in serum, serum samples from eight patients were spiked with GHB at a concentration of 287 mg/L. The samples had a mean concentration of 281 mg/L, a CV of 2.7% and an accuracy at 98%.

## Case History

An otherwise healthy 81-year-old woman was found unconscious in the middle of the night on her bathroom floor. The following were noted in the ambulance journal: Glasgow coma scale (GCS) 3, diameter of right and left pupil were 2 mm, blood glucose 9.9 mmol/L, oxygen saturation 96%, heart frequency 62 beats/min, respiratory frequency 20 breaths/min, systolic blood pressure 127 mmHg and body temperature 35.3°C. The paramedics suspected an acute cerebral event. In addition, a concern of intoxication was raised since several tablets of quetiapine (Seroquel^®^) were found beside the patient. She was unconscious but vitally stable during transport to the hospital.

Upon admission, naloxone 0.4 mg and flumazenil 0.3 mg were administered intravenously without any response. An additional 0.4 mg of naloxone was administered. The patient responded with movements of the extremities and with movements in response to pain stimulation. This was interpreted as an effect of naloxone. The patient was therefore administered another 0.4 mg naloxone. She was given a total amount of 1.2 mg of naloxone.

Shortly after admission, a computed tomography with angiography of the central nervous system was unremarkable. Biochemical analysis of the blood did not show signs of inflammation or infection. Ethanol and quetiapine were not detected in the serum. The patient’s next of kin contacted the hospital and informed the attending physician that a relative of the patient, who has a substance use disorder, was living with the patient. Due to the negative findings of the supplemental examinations, the possible effect of naloxone and the information provided by next of kin, a preliminary diagnosis of intoxication by opioids was suspected. The patient was admitted to the intermediate ward.

The level of consciousness was fluctuating the first hours after admission. She woke up and called for help 6 hours after she was found unconscious at home. She did not understand where she was or why she was hospitalized. During the next hours, communication with the patient was difficult. She repeated questions, and she did not remember having children or that she was living with a relative. It is noted in the medical journal that she had a tremor, was restless and had one episode of vomiting. She denied having suicidal thoughts and denied any drug intake prior to the incident. She had no recollection of what had happened prior to the incident but remembered that she woke up at home sometime during the night due to a fire alarm going off. This was probably prior to losing consciousness.

A psychiatric evaluation was required. The psychiatrist concluded that the patient neither seems to have severe depression or psychotic symptoms nor was there any concern of suicidal intent. In the evening, around 17 hours after she was found at home, she responded adequately to questions and seemed to be back to her habitual state.

Because of the suspected intoxication, a urine sample was obtained 27 hours after admittance. A preliminary drug screening with immunoassay detected no drugs. Further analysis with liquid chromatography--quadrupole time-of-flight mass spectrometry (LC--QTOF-MS) was performed. Naloxone was the only detected drug. The routine analytical method included benzodiazepines and z-hypnotics, opioids and central stimulating agents.

The clinician still suspected a possible case of intoxication and therefore required a GHB in urine. GHB was detected at a concentration of 374 mg/L, using LC--MS-MS. On the basis of this, it was decided to analyze the serum sample from admittance, using the same analytical method. The serum sample contained GHB in a concentration of 222 mg/L.

The patient was notified of the GHB result on telephone since the result was available after she was discharged. Upon the notification, she admitted that she drank from the same soda bottles as her relative from time to time. Norwegian media have previously reported that soda bottles could be used to store GHB (5), and this could be the source of the intoxication in the present case.

## Discussion

To our knowledge, this is the first case report of an elderly patient intoxicated with GHB. The pharmacodynamics and PK of GHB in elderly patients are not outlined in the literature.

When it comes to pharmacodynamics, the clinical presentation is consistent with reported intoxications in younger adults. Common signs of GHB intoxication in younger adults include both pathophysiological manifestations like hypotension, respiratory depression, miosis, bradycardia and hypothermia as well as unspecific symptoms like disorientation, dizziness, nausea, euphoria, amnesia and hallucinations. Somnolence and low GCS score are also common symptoms; some studies have shown that >50% of users of GHB have experienced unconsciousness. Rapid recovery is the general rule when it comes to acute GHB intoxication. This reflects the short elimination half-life of GHB (6). The elderly patient presented with bradycardia, hypothermia, miosis and unconsciousness and proceeded to show clear symptoms of anterograde amnesia after regaining consciousness, all consistent with the reported signs of GHB intoxication for younger adults. The effects of GHB are usually manifested within 15 min after administration and persist for ∼3 hours on average (7). We do not have confirmation of the time of intake, but her relative reported to have observed her being aware and oriented only minutes before he found her on the bathroom floor. We therefore assume that the intake of GHB was made shortly before she was found unconscious. She regained consciousness after a few hours, as one would expect in a case of GHB intoxication.

Several publications have reported the detection time of GHB in urine in adults to be ∼12 hours (8, 9). To our knowledge, this is the first case where GHB has been detected in urine >24 hours after ingestion. Possible explanations for the extended detection time could be the combination of changes in the PK due to old age and, based on the high serum concentration, probably a massive dose ingested. GHB shares many of the same characteristics as ethanol (3), in which PK changes due to old age have been shown to contribute to higher ethanol concentrations and longer elimination time (10). Ethanol is subject to first-pass metabolism that is shown to be reduced with increasing age, due to atrophy in the gastric mucosa, reduced number of hepatocytes, reduced liver blood flow and reduced enzymatic activity. In theory, all these factors could contribute to the extended detection of GHB. GHB dehydrogenase is the most important enzyme in the metabolism of GHB, catalyzing the conversion of GHB to succinic semialdehyde. The metabolism seems to be saturable, and an age-related reduction in the activity of the enzyme would, therefore, possibly prolong the half-life and detection time. Hepatic metabolism is likely the primary route, meaning that a reduced number of hepatocytes and reduced liver blood flow would in theory contribute to a prolonged detection time (11). Whether there is an enzymatic activity in the gastrointestinal tract that could be reduced by age-related atrophy in the gastric mucosa is yet to be elucidated. Furthermore, GHB, as ethanol, is distributed to the total body water. One would therefore expect the volume of distribution (Vd) to be lower in females than in males and lower in the elderly (due to less water per kilogram body weight) (6). This could contribute to a higher Cmax but not to a longer half-life and detection time.

The serum concentration of 222 mg/L was quantified in a sample obtained shortly after admittance at the hospital. The GHB absorption phase is reported to be ∼30–50 min (6). The sample was drawn about an hour after she was found unconscious, meaning that the serum concentration probably was close to Cmax (12). The GHB concentration is in a range where one would expect severe symptoms of intoxication. The mean and range of GHB concentrations in patients admitted to a hospital for overdosing with GHB were 137  and 29–490 mg/L, respectively (13). In an Australian study on deaths related to GHB, the median blood concentration postmortem was 210 mg/L (the blood plasma ratio is estimated to be 1.2 (14)). In most of the cases, other drugs were present as well. The median urinary concentration in the same study was 297 mg/L (15). Busardo and Jones reported postmortem GHB concentrations between 30 and 9,200 mg/L, with a mean concentration of 640 mg/L in deaths related to intoxication (2).

The GHB concentration in urine was high, considering that the sample was obtained 27 hours after admittance. The mean Cmax after the administration of therapeutic doses has been shown to be somewhat lower, with a Tmax between 1 and 3 hours (16). However, a considerable individual variability was shown by Brenneisen et al. (8). Unfortunately, blood sampling was not performed around the same time as the urine sample was obtained. Symptoms of impairment were not observed although a systematic examination was not performed. It is previously reported that GHB might not be detected in blood, whereas a high concentration could be present in bladder urine postmortem (2).

A rapid elimination, short detection times in common matrices and endogenous presence are some of the characteristics making GHB an analytical challenge (17). In addition, when using LC--MS, GHB needs to be analyzed in a negative ion mode. The LC--QTOF-MS technology does not allow ion switching during an analysis. Hence, at our laboratory, GHB is allocated to a separate method together with other analytes demanding a negative ion mode and has to be requisitioned separately when a drug screening is required. GHB is often not included in routine screenings for drugs of abuse in urine (18). However, this case report illustrates that in clinical situations where intoxication with an unknown substance is suspected, GHB analysis should be considered.

The positive GHB result was available after the patient was discharged and therefore had no impact on the emergency treatment. The result did, however, have consequences for the follow-up of the patient. When she was discharged, she was given a driving ban because of the lack of an explanation for her illness. Detecting GHB resulted in a reversal of the driving ban.

In conclusion, this case report indicates that GHB might have a longer elimination time in elderly people and that, although sampling should be made as early as possible, even a urine sample obtained >24 hours after the time of GHB intake could yield a positive result. The case also underlines the need to consider intoxication as a differential diagnosis in elderly patients with reduced consciousness.

## Data Availability

The authors confirm that the data supporting the findings of this study are available within the article.
